# Testimonials and Informational Videos on Branded Prescription Drug Websites: Experimental Study to Assess Influence on Consumer Knowledge and Perceptions

**DOI:** 10.2196/jmir.7959

**Published:** 2018-01-23

**Authors:** Helen W Sullivan, Amie C O'Donoghue, Jennifer Gard Read, Jacqueline B Amoozegar, Kathryn J Aikin, Douglas J Rupert

**Affiliations:** ^1^ US Food and Drug Administration Silver Spring, MD United States; ^2^ RTI International Research Triangle Park, NC United States

**Keywords:** Internet, marketing, drug prescriptions, risk

## Abstract

**Background:**

Direct-to-consumer (DTC) promotion of prescription drugs can affect consumer behaviors and health outcomes, and Internet drug promotion is growing rapidly. Branded drug websites often capitalize on the multimedia capabilities of the Internet by using videos to emphasize drug benefits and characteristics. However, it is unknown how such videos affect consumer processing of drug information.

**Objective:**

This study aimed to examine how videos on prescription drug websites, and the inclusion of risk information in those videos, influence consumer knowledge and perceptions.

**Methods:**

We conducted an experimental study in which online panel participants with acid reflux (n=1070) or high blood pressure (n=1055) were randomly assigned to view 1 of the 10 fictitious prescription drug websites and complete a short questionnaire. On each website, we manipulated the type of video (patient testimonial, mechanism of action animation, or none) and whether the video mentioned drug risks.

**Results:**

Participants who viewed any video were less likely to recognize drug risks presented only in the website text (*P*≤.01). Including risk information in videos increased participants’ recognition of the risks presented in the videos (*P*≤.01). However, in some cases, including risk information in videos decreased participants’ recognition of the risks *not* presented in the videos (ie, risks presented in text only; *P*≤.04). Participants who viewed a video without drug risk information thought that the website placed more emphasis on benefits, compared with participants who viewed the video with drug risk information (*P*≤.01). Compared with participants who viewed a video without drug risk information, participants who viewed a video with drug risk information thought that the drug was less effective in the high blood pressure sample (*P*=.03) and thought that risks were more serious in the acid reflux sample *(P*=.01). There were no significant differences between risk and nonrisk video conditions on other perception measures (*P*>.05). In addition, we noted a few differences among the types of videos.

**Conclusions:**

Including risks in branded drug website videos may increase in-video risk retention at the expense of text-only risk retention.

## Introduction

### Background

Direct-to-consumer (DTC) marketing of prescription drugs is correlated with increases in consumer drug spending, prescription drug use, and prescription requests [1-6]. However, studies have revealed mixed findings as to whether DTC marketing leads to more informed decisions by consumers [7-9]. DTC marketing on the Internet is rapidly growing [10,11] with a 648% increase in spending on Internet promotion between 2001 and 2010 [12]; therefore, it is critical to understand how this medium may affect outcomes related to informed decision making.

Pharmaceutical companies use a number of different Internet activities, most commonly to promote products and communicate with consumers [13]. To this end, most leading pharmaceutical companies maintain two types of websites: websites with corporate business information and websites promoting specific drugs or medications, known as branded drug websites (eg, DrugX.com). Branded drug websites typically provide information about the branded drug, the disease or medical condition, support measures, and ways to locate physicians and pharmacists [14]. Branded drug websites often use videos as a marketing tool to emphasize a promoted drug’s benefits and characteristics [14-18]. These videos may share features with DTC television advertising. For instance, DTC television advertisements frequently include factual information and positive emotional appeals, often in the form of testimonials [19]. However, consumers may react differently to videos in Internet promotion [20]. One way in which videos on websites differ from television advertisements is that these videos are often presented along with text on the website, creating a mix of dynamic and static formats. The movement in the video could increase attention to the video, thereby causing greater recall of the information in the video and perhaps influencing the perceptions of the risks or the benefits. Previous research on the influence of dynamic videos is mixed [21], but a meta-analysis [22] found a small benefit for dynamic versus static images when learning information.

### Testimonial Videos

Branded drug websites have sometimes showcased video testimonials of expert medical sources as well as patients who have been treated successfully with the promoted drug [15,16]. Previous research has not examined testimonials within the context of DTC marketing; however, studies in other health-related areas have found mixed evidence about how patient testimonials affect individuals’ perceptions of their disease risk. Some studies have found that testimonials heighten consumer risk perceptions for issues such as human papillomavirus (HPV), human immunodeficiency virus (HIV), and other sexually transmitted diseases [23,24]. However, other studies have found that testimonials did not change consumer risk perceptions on these issues [25]. Trevana and colleagues reviewed 3 articles that used narratives to convey risk information and concluded that patient testimonials should be used with caution because they can have unpredictable effects on risk perceptions [26]. In addition, another study concluded that the use of patient testimonials in decision aids should be avoided until their impact is better understood, because there are concerns that stories have the potential to bias patients’ decisions [27]. Given these mixed findings, we examined the role of video testimonials on branded drug websites, where the understanding of drug risks and benefits is a critical part of an informed discussion with a health care provider.

### Informational Videos

Another strategy to promote branded drugs on the Internet is the use of videos that provide information about the product. Although previous studies have examined the presentation of information about topics ranging from astrophysics [28] to pulley systems [21], we are unaware of any study that has examined the presentation of prescription drug information on branded drug websites. DTC promotion is replete with technical information that must be conveyed to adequately represent the benefits and risks of the product, according to US Food and Drug Administration (FDA) regulations [29]. Thus, we examined the presence of a dynamic informational video on the website describing the drug’s mechanism of action to determine whether this presentation influenced viewers’ understanding of the promoted drug’s benefits and risks.

### Research Questions

FDA regulations state that prescription drug promotion should include a fair balance of information about the benefits and risks of promoted products, in terms of both content and presentation [29]. The regulations further specify that important risk information should be presented in at least the audio, or in both the audio and visual of broadcast advertisements. All prescription drug promotions should be truthful, balanced, and nonmisleading, regardless of the media in which that promotion occurs [30]. However, questions remain about how best to achieve “fair balance” in Internet DTC promotion [31-34]. The purpose of this study was to examine how videos on prescription drug websites—and the content of those videos—influence consumers’ knowledge, perceptions, and intentions related to the advertised drug.

We had 2 overarching research questions: (1) how does the presence of Web videos (testimonials and informational videos) influence consumer knowledge and perceptions of drugs? and (2) how does including risk information in Web videos influence consumer knowledge and perceptions of drugs? We hypothesized that the presence, versus absence, of Web videos would lead to greater retention of benefit information, and therefore higher drug efficacy perceptions and greater intentions to search for information about the drug. This hypothesis is based on the expectation that video presentations draw attention and that the focus of videos is often benefit information (eg, centered on a patient attesting to the drug effectiveness). Accordingly, we also hypothesized that the presence, versus absence, of Web videos would lead to less retention of the risk information, and therefore lower drug risk perceptions. In addition, we hypothesized that videos including risk information, compared with videos without risk information, would lead to greater retention of the risk information, and therefore higher drug risk perceptions and lower intentions to search for information about the drug. We did not have hypotheses about potential differences between testimonials and informational videos; however, because we randomly assigned participants to the type of video they viewed, we tested for any differences between these conditions. Finally, we explored whether including a video and whether including risk information in the video influenced perceptions of the website itself.

## Methods

### Study Design

**Figure 1:**
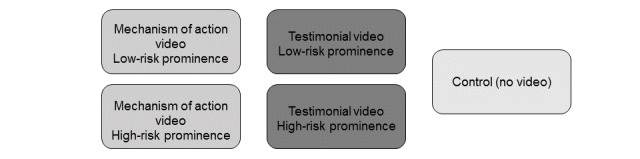
Experimental conditions.

We conducted the study with consumers with acid reflux and consumers with high blood pressure and exposed them to a mock drug website advertising a fictitious drug (Fentiva or Plistaz) designed to treat their respective condition. We chose these medical conditions because they affect a large number of people and represent both symptomatic (acid reflux) and asymptomatic (high blood pressure) conditions. We manipulated the type of video participants viewed and the prominence of the risk information on the websites. All websites contained the benefit and risk information in the text. We manipulated whether the websites presented a video of a personal testimonial, an informational mechanism of action video, or no video at all (control group). In the video conditions, we also manipulated the prominence of the risk information by including some risk information (high-risk prominence) or by not including some risk information (low-risk prominence) in the video. Experimental conditions are presented in Figure 1. To ensure that our fictitious websites were realistic, we reviewed actual prescription drug websites and consulted FDA’s Office of Prescription Drug Promotion. We designed the fictitious websites to mirror real-life prescription drug websites in structure (eg, homepage plus 2 subpages), content (eg, drug benefits and risks and tips for disease management), and design (eg, consistent banner and heading across all pages and photos of patients or caregivers). The study was approved by the relevant institutional review boards.

### Participants

The sampling frame was the GfK Custom Research North America KnowledgePanel, a probability-based online consumer panel based on a representative random sample of US adults. Panelists were randomly selected and invited to participate in the study if they were aged 18 years or older, self-reported to the panel that they were medically diagnosed with acid reflux or high blood pressure, did not participate in the study pretest, were capable of viewing and listening to websites on a desktop computer or device, had broadband Internet access, and used a device with Adobe Flash Player software. We randomly assigned participants to 1 of the 5 experimental arms within each illness group and confirmed their self-reported medical diagnosis. For acid reflux, we invited a total of 2226 panelists to participate in the study. Out of these panelists, 1774 responded and 1070 completed the study. For high blood pressure, we invited a total of 2020 panelists to participate in the study. Out of these panelists, 1559 responded and 1055 completed the study. A summary of participants’ demographic characteristics is presented in Table 1.

### Procedure

After completing the screening questions, participants were instructed to click a hyperlink to open the study website in a new window (675×1064 pixels). In addition, participants were instructed to turn up the volume and watch any videos. Exposure to the website was forced (ie, participants were not able to proceed to the questionnaire without clicking the hyperlink and landing on the website). Each website included a homepage and 2 subpages. Participants were allowed to navigate back and forth among them without time restrictions. Internet browser controls were removed from the stimuli window to simplify navigation.

**Figure 2:**
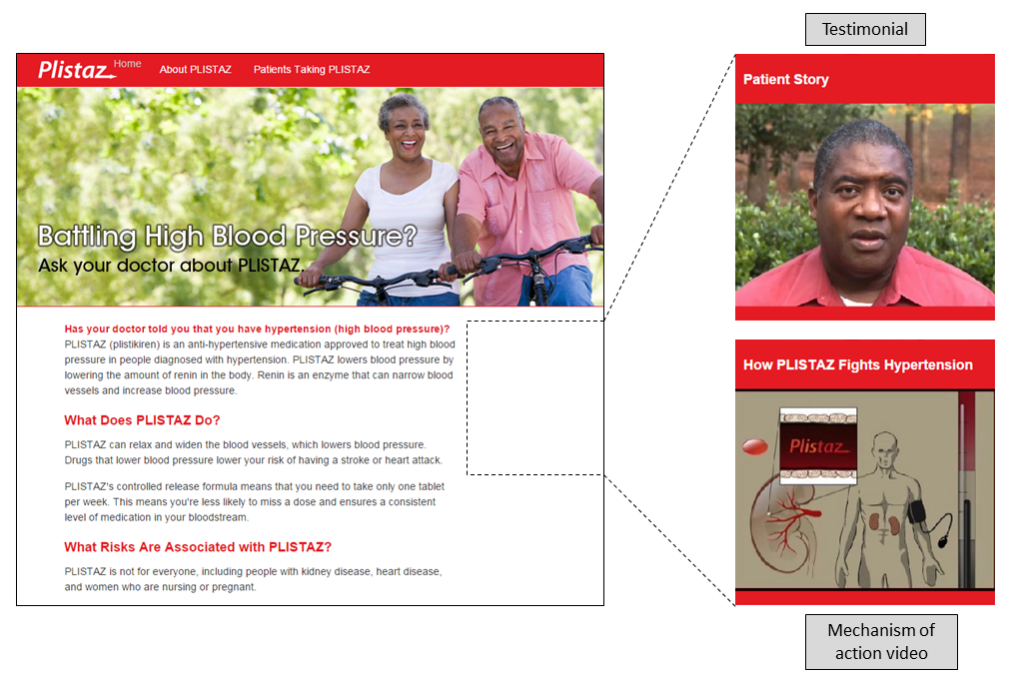
Example of study stimuli.

In all conditions, the website presented both the drug’s risk and benefit information as text. In the control condition, no video was present on the website. In the testimonial condition, the website included a video featuring an actor depicting a patient who described how the drug worked for them (including treating acid reflux or lowering blood pressure). In the mechanism of action video condition, the website included a dynamic animated video depicting how the drug mechanism treats the illness (including treating acid reflux or lowering blood pressure). In the high-risk prominence testimonial condition, the “patient” stated some of the risk information. In the high-risk prominence mechanism of action videos, the voiceover stated some of the risk information while the risks were presented dynamically through text or icons. The risk information included in the high-risk prominence conditions was identical in the testimonials and mechanism of action videos. The website layout is depicted in Figure 2.

**Table 1:** Demographic characteristics of the participants.

Demographic characteristics		Acid reflux sample, weighted (N=1070) n (%)	High blood pressure sample, weighted (N=1055) n (%)
**Sex**			
	Male	449 (41.96)	505 (47.87)
	Female	621 (58.04)	550 (52.13)
**Age, years**			
	18-24	22 (2.06)	6 (0.57)
	25-34	69 (6.45)	26 (2.46)
	35-44	145 (13.55)	94 (8.91)
	45-54	212 (19.81)	188 (17.82)
	55-64	274 (25.61)	313 (29.67)
	65-74	249 (23.27)	310 (29.38)
	75+	99 (9.25)	118 (11.18)
**Race or ethnicity**^a^			
	White	925 (86.45)	838 (79.43)
	Black	97 (9.06)	194 (18.39)
	Other	105 (9.81)	80 (7.58)
	Hispanic	88 (8.22)	93 (8.82)
**Education**			
	Less than high school	52 (4.86)	50 (4.74)
	High school graduate	419 (39.16)	408 (38.67)
	Some college	315 (29.44)	305 (28.91)
	College degree or higher	284 (26.54)	292 (27.68)

^a^There is some overlap between categories as Hispanics are also counted in the 3 race categories.

We programmed the videos to play automatically, thus forcing exposure to the manipulations. No control bar was available; participants could not stop or mute the video once it began playing, although they could close the website whenever they wanted. Participants could replay the video as many times as desired. Once participants closed the stimuli window and continued to the questionnaire, they were unable to view the website again.

### Measures

We used Web logs to track participants’ interaction with the fictitious drug websites and translated these logs into navigation variables that could be used to analyze participants’ behavior, including two measures of video exposure: (1) whether participants were fully exposed or partially exposed to the video and (2) whether or not they replayed the video.

We measured risk recall by asking participants to list the risks of the drug in an open-ended text box. For the acid reflux sample, we created a measure of risk recall by coding whether participants reported the risks presented in the video: nausea, headache, stomach pain, diarrhea, constipation, or fractures (0-6). For the high blood pressure sample, we created a measure of risk recall by coding whether participants reported the side effects (diarrhea, rash, or cough) and limitations (that the drug could not be taken if pregnant, used as a salt substitute, used with salt, or used while drinking alcohol) presented in the video (0-7).

To measure risk recognition, we presented participants with two risks that appeared in the video and in the website text, two risks that appeared in the website text only, and four filler statements. Participants indicated whether each statement was mentioned on the website as a risk of taking the drug. For the acid reflux sample, we measured whether they recognized the risks presented in the video [fracture risk (0-1) and nausea risk (0-1)] and the risks presented in the website text only [warnings and precautions regarding special liver tests (0-1) and women who are nursing (0-1)]. Note that although the videos did not include the warning about special liver tests, they did say to tell your doctor if you have liver disease. For the high blood pressure sample, we measured whether they recognized the risks presented in the video [diarrhea risk (0-1) and salt-intake warning (0-1)] and the risks presented in the website text only [fetal risk (0-1) and angioedema warning (0-1)].

We measured benefit recall by asking participants to list the benefits of the drug in an open-ended text box. For the acid reflux sample, we created a measure of benefit recall by coding whether participants reported that the drug could relieve acid reflux [including terms such as heartburn and GERD (gastroesophageal reflux disease); (0-1)]. For the high blood pressure sample, we created a measure of benefit recall by coding whether participants reported that the drug could lower blood pressure (0-1).

To measure benefit recognition, we presented participants with a list of statements about the drug. Participants indicated whether each statement was mentioned on the website as a benefit of taking the drug. For the acid reflux sample, we measured whether the participants recognized the benefit of the drug, “Fentiva can provide relief from heartburn” (0-1). For the high blood pressure sample, we measured whether the participants recognized the benefit of the drug, “Plistaz lowers blood pressure by reducing the amount of renin in the body” (0-1).

Perceived drug risk was measured by 2 items that assessed participants’ thoughts on how many people would have side effects out of 100 people who take the drug (*likelihood*; open-ended item with values ranging from 0 to 100) and how serious the side effects would be for them (*magnitude*; 1=not at all serious, 6 = very serious). Perceived drug efficacy was measured by 2 items that assessed participants’ thoughts on how many people would benefit out of 100 people who take the drug (*likelihood*; open-ended item with values ranging from 0 to 100) and how effective the drug would be for them (*magnitude*; 1=would help very little to 6=would help a lot). Perceived balance of drug benefits and risks was measured by asking participants how they would rate the drug on its balance of risks and benefits (1=risks outweigh benefits to 7=benefits outweigh risks).

We measured two types of behavioral intentions: the intention to interact with one’s physician and the intention to seek additional information about the drug on the Internet. For the physician intention measure, we averaged 3 items that assessed how likely participants were to talk to their doctor about the drug, ask their doctor for a sample of the drug, and ask their doctor to prescribe the drug (acid reflux alpha=.94; high blood pressure alpha=.93). For the information-seeking intention measure, we averaged 3 items that assessed how likely participants were to look for information about the drug on medical websites, look for information on the Internet about people’s experience with the drug, and print information from the drug website (acid reflux alpha=.90; high blood pressure alpha=.90). All intention items used the same scale (1=very unlikely to 5=very likely).

We also asked participants about the website itself. We measured website skepticism with the average of 2 items that assessed whether participants thought the information on the websites was true (acid reflux alpha=.66; high blood pressure alpha=.71). Perceived balance of website benefits and risks was measured by asking participants whether they thought the website placed more emphasis on risks or benefits of the drug (1=more emphasis on risks to 7=more emphasis on benefits).

### Data Analysis

Weighting was used to account for the underrepresentation of minority groups and other types of sampling and survey errors. We transformed *perceived drug efficacy likelihood* (by squaring it) and *perceived drug risk likelihood* (by using the natural log), resulting in approximately normal distribution of the data in the two illness populations. We conducted all hypothesis tests using the transformed variables.

We conducted one-way analysis of variances (ANOVAs) to test the relationship between video type condition (testimonial, mechanism of action, and control) and the risk recall, perception, intention, and skepticism measures. If the effect was significant at *P*<.05, we performed pairwise comparisons by comparing the testimonial and mechanism of action video type conditions with the control condition, using a Bonferroni-adjusted threshold of *P*=.025. We conducted two-way ANOVAs to test the relationship between the video type condition (testimonial and mechanism of action) and risk prominence (low and high) and the risk recall, perception, intention, and skepticism measures. We conducted two sets of logistic regressions for risk recognition and benefit recall and retention: one examining the video type conditions compared with the control condition and one examining the main effects and interaction of video type condition and risk prominence.

## Results

### Video Exposure

#### Acid Reflux and High Blood Pressure

All participants were exposed to the video, and most of them viewed the entire video (94.8% in the acid reflux sample and 98.6% in the high blood pressure sample). Only a few participants replayed the video (7.5% in the acid reflux sample and 6.8% in the high blood pressure sample).

### Retention of Drug Risk Information

#### Acid Reflux

We tested the effect of risk prominence and video type condition on risk recall. We found that participants in the high-risk prominence condition recalled more risks compared with participants in the low-risk prominence condition (*F*_1,860_=4.00, *P*=.046, *d*=.14; Table 2).

We also tested the effect of risk prominence and video type condition on the risk recognition measures. We found a significant effect of risk prominence on two of the risk recognition measures, and a significant interaction on a third measure. Compared with participants in the low-risk prominence condition, participants in the high-risk prominence condition were more likely to recognize the risk of fracture presented in the video and the risk of special liver tests that was alluded to in the video but presented only in the website text (*F*_1,860_=6.54, *P*=.01, *d*=.17 and *F*_1,860_=7.88, *P*=.01, *d*=.19, respectively). There was a significant interaction for the nursing warning presented only in the website text (*F*_1,860_=10.38, *P*=.001, *d*=.22). In the mechanism of action conditions, 83.8% (standard error [SE] 3.0) of participants in the high-risk prominence condition and 75.2% (SE 4.7) in the low-risk prominence condition recognized the nursing warning; however, this was reversed in the testimonial conditions (high-risk prominence=64.1% [SE 5.2] and low-risk prominence=83.1% [SE 3.6]).

**Table 2:** Weighted percentages and means (standard errors) by risk prominence.

Risk retention variables		AR^a^ sample		HBP^b^ sample	
		Low-risk prominence (n=426)	High-risk prominence (n=435)	Low-risk prominence (n=419)	High-risk prominence (n=404)
Risk recall^c^, mean (SE)		1.80 (0.12)^d^	2.13 (0.11)	1.02 (0.09)	1.12 (0.08)
**Risk recognition: video, % (SE)**					
	AR fracture risk and HBP diarrhea risk	77.5 (3.4)^d^	88.4 (2.5)	66.6 (3.7)^d^	86.9 (2.7)
	AR nausea risk and HBP salt intake warning	77.5 (3.2)	77.2 (3.1)	68.0 (3.3)	71.1 (3.6)
**Risk recognition: text, % (SE)**					
	AR liver warning and HBP fetal risk	65.4 (3.7)^d^	79.3 (3.2)	69.3 (3.5)^d^	58.7 (3.7)
	AR nursing warning and HBP angioedema warning	79.1 (3.0)	73.9 (3.1)	63.0 (3.9)^d^	49.0 (3.7)
Benefit recall, % (SE)		44.2 (3.9)	38.3 (3.5)	70.1 (3.6)	62.3 (3.7)
Benefit recognition, % (SE)		85.6 (2.7)	82.7 (2.8)	88.4 (2.7)	80.3 (3.3)

^a^AR: acid reflux.

^b^HBP: high blood pressure.

^c^Risk recall: 0-6 correct in the AR sample and 0-7 in the HBP sample.

^d^Significantly different from the high-risk prominence condition, *P*<.05.

On comparing the video type conditions with the control condition on risk recall and risk recognition, we found effects for one risk recognition measure. Compared with participants in the mechanism of action and testimonial conditions, participants in the control condition were more likely to recognize the nursing warning presented only in the website text (*F*_1,1069_=6.05, *P*=.01, *d*=.15 and *F*_1,1069_=12.18, *P*=.001, *d*=.21, respectively).

The risk prominence and video type conditions did not significantly differ on the risk recognition measure regarding the nausea risk presented in the video (*P*>.05).

#### High Blood Pressure

We tested the effect of risk prominence and video type condition on risk recall. We found that participants in the mechanism of action condition recalled more risks compared with participants in the testimonial condition (*F*_1,822_=5.04, *P*=.03, *d*=.16; Table 3).

We also tested the effect of risk prominence and video type condition on the risk recognition measures. We found a significant effect of risk prominence on two of the risk recognition measures. Compared with participants in the low-risk prominence condition, participants in the high-risk prominence condition were more likely to recognize the risk of diarrhea presented in the video (*F*_1,822_=17.22, *P*<.001, *d*=.29). However, they were *less* likely to recognize the fetal and angioedema risks presented only in the website text (*F*_1,822_=4.30, *P*=.04, *d*=.14 and *F*_1,822_=6.39, *P*=.01, *d*=.18, respectively).

On comparing the video type conditions with the control condition on risk recall and risk recognition, we found effects for one risk recognition measure. Participants in the control condition were more likely to recognize the angioedema risk presented only in the website text compared with participants in the testimonial condition (*F*_1,1054_=12.33, *P*<.001, *d*=.22).

The risk prominence and video type condition did not significantly differ on the risk recognition measure regarding the salt intake risk presented in the video (*P*>.05).

### Retention of Drug Benefit Information

#### Acid Reflux

We tested the effect of risk prominence and video type condition on benefit recall and benefit recognition. We found no significant effects for benefit recall (*P*>.05). We found significant effects for benefit recognition when we compared the video type conditions. Specifically, participants in the testimonial and control conditions were more likely to recognize the drug’s benefit compared with participants in the mechanism of action conditions (*F*_1,860_=15.22, *P*<.001, *d*=.26 and *F*_1,1069_=11.74, *P*=.001, *d*=.21, respectively).

#### High Blood Pressure

We tested the effect of risk prominence and video type conditions on benefit recall and benefit recognition. We found significant effects for benefit recall and benefit recognition when we compared the video type conditions. Participants in the mechanism of action condition were more likely to recall the drug’s benefit compared with participants in the testimonial and control conditions (*F*_1,822_=5.27, *P*=.02, *d*=.16 and *F*_1,1054_=7.50, *P*=.01, *d*=.17, respectively). They were also more likely to recognize the drug’s benefit compared with participants in the testimonial condition (*F*_1,822_=6.01, *P*=.01, *d*=.17).

**Table 3:** Weighted percentages and means (standard errors) by video type condition.

Risk retention variables		AR^a^ sample			HBP^b^ sample		
		Mechanism of action (n=430)	Testimonial (n=431)	Control (n=209)	Mechanism of action (n=411)	Testimonial (n=412)	Control (n=232)
Risk recall^c^, mean (SE)		1.90 (0.11)	2.03 (0.12)	1.91 (0.15)	1.20^d^ (0.09)	0.94 (0.08)	1.13 (0.11)
**Risk recognition: video, % (SE)**							
	AR fracture risk and HBP diarrhea risk	83.7 (3.0)	82.4 (3.1)	88.8 (3.7)	79.1 (3.0)	74.0 (3.6)	80.6 (4.0)
	AR nausea risk and HBP salt intake warning	75.0 (3.4)	79.6 (2.9)	80.1 (4.5)	68.9 (3.3)	70.1 (3.6)	74.4 (4.4)
**Risk recognition: text, % (SE)**							
	AR liver warning and HBP fetal risk	68.6 (3.7)	76.2 (3.1)	66.2 (5.3)	63.8 (3.5)	35.6 (3.8)	75.9 (4.3)
	AR nursing warning and HBP angioedema warning	79.5 (2.8)^e^	73.5 (3.3)^e^	89.9 (2.6)	61.2 (3.7)	51.1 (3.9)^e^	74.3 (4.7)
Benefit recall, % (SE)		41.7 (3.6)	40.7 (3.8)	55.1 (5.3)	72.1 (3.3)^d,e^	60.5 (3.9)	55.3 (5.3)
Benefit recognition, % (SE)		77.4 (3.1)^d,e^	90.9 (2.1)	93.1 (2.3)	89.8^d^ (2.3)	79.1 (3.6)	81.0 (4.0)

^a^AR: acid reflux.

^b^HBP: high blood pressure.

^c^Risk recall: 0-6 correct in the AR sample and 0-7 in the HBP sample.

^d^Significantly different from the testimonial condition, *P*<.05.

^d^Significantly different from the control condition; Bonferroni-adjusted for two comparisons with the control condition, *P*<.025.

**Table 4:** Weighted means (standard errors) by risk prominence.

Dependent variables		Acid reflux sample, mean (SE)		High blood pressure sample, mean (SE)	
		Low-risk prominence	High-risk prominence	Low-risk prominence	High-risk prominence
**Perceived drug risk**					
	Likelihood^a^	33.11 (1.92)	31.93 (1.69)	33.34 (1.83)	37.29 (1.89)
	Magnitude^b^	3.46^c^ (0.09)	3.76 (0.08)	3.75 (0.09)	3.73 (0.08)
**Perceived drug efficacy**					
	Likelihood^a^	70.66 (1.50)	71.43 (1.47)	64.48 (1.80)	65.25 (1.69)
	Magnitude^d^	4.84 (0.08)	4.95 (0.07)	4.79^c^ (0.08)	4.52 (0.09)
Perceived balance of drug benefits and risks^e^		4.51 (0.11)	4.42 (0.11)	4.49 (0.08)	4.33 (0.10)
**Intention^f^**					
	Physician interaction	2.88 (0.09)	2.74 (0.09)	2.55 (0.10)	2.29 (0.10)
	Search on the Internet	2.50 (0.09)	2.39 (0.08)	2.38 (0.10)	2.12 (0.09)
Website skepticism^g^		3.49 (0.09)	3.45 (0.08)	3.77 (0.08)	3.75 (0.10)
Perceived balance of website benefit and risk information^h^		5.16^c^ (0.10)	4.72 (0.10)	5.14^c^ (0.10)	4.78 (0.10)

^a^Perceived drug risk and efficacy likelihood: 0-100 people. Although transformations of perceived drug risk and efficacy likelihood were used in analyses, the untransformed weighted means are presented here for the ease of interpretation.

^b^Perceived drug risk magnitude: 1 (not at all serious) to 6 (very serious).

^c^Significantly different from the high-risk prominence condition, *P*<.05.

^d^Perceived drug efficacy magnitude: 1 (help a little) to 6 (help a lot).

^e^Perceived balance of drug benefits and risks: 1 (risks outweigh benefits) to 7 (benefits outweigh risks).

^f^Intention: 1 (very unlikely) to (5=very likely).

^g^Website skepticism: 1 (extremely unlikely) to 7 (extremely likely).

^h^Perceived balance of website benefit and risk information: 1 (more emphasis on risks) to 7 (more emphasis on benefits).

### Perceived Drug Efficacy

#### Acid Reflux

We tested the effect of risk prominence and video type condition on the perceived drug efficacy measures. We found no significant effects for perceived drug efficacy likelihood or magnitude (*P*>.05; Tables 4 and 5).

#### High Blood Pressure

We tested the effect of risk prominence and video type condition on the perceived drug efficacy measures. We found a significant effect of risk prominence on one of the measures. Specifically, participants in the low-risk prominence condition thought that the drug would work better compared with participants in the high- risk prominence condition (*F*_1,810_=4.60, *P*=.03, *d*=.15). All other effects for the perceived drug efficacy measures were nonsignificant (*P*>.05).

### Perceived Drug Risk

#### Acid Reflux

We tested the effect of risk prominence and video type condition on perceived drug likelihood and magnitude. For perceived drug magnitude, we found a significant effect when comparing video type conditions with the control condition and a significant effect of risk prominence. Participants in the control condition thought that the drug’s side effects and negative outcomes would be more serious compared with participants in the mechanism of action condition (*F*_1,1048_=7.47, *P*=.01, *d*=.17). In addition, participants in the high-risk prominence condition thought that the drug’s side effects and negative outcomes would be more serious compared with participants in the low-risk prominence condition (*F*_1,841_=6.40, *P*=.01, *d*=.17). We found no significant effects for perceived drug risk likelihood (*P*>.05).

#### High Blood Pressure

We tested the effect of risk prominence and video type condition on perceived drug likelihood and magnitude. We found no significant effects (*P*>.05).

### Perceived Balance of Drug Benefits and Risks

#### Acid Reflux

We tested the effect of risk prominence and video type condition on the perceived balance of drug benefits and risks. We found no significant effects (*P*>.05).

**Table 5:** Weighted means (standard errors) by video type condition.

Dependent variables		Acid reflux sample, mean (SE)			High blood pressure sample, mean (SE)		
		Mechanism of action	Testimonial	Control	Mechanism of action	Testimonial	Control
**Perceived drug risk**							
	Likelihood^a^	34.40 (1.95)	30.67 (1.62)	36.08 (2.69)	37.25 (1.90)	33.21 (1.78)	35.98 (2.49)
	Magnitude^b^	3.52^c^ (0.08)	3.70 (0.10)	3.93 (0.13)	3.72 (0.09)	3.76 (0.09)	3.89 (0.12)
**Perceived drug efficacy**							
	Likelihood^a^	72.38 (1.34)	69.70 (1.61)	74.97 (2.09)	66.48 (1.40)	63.20 (2.02)	64.23 (1.98)
	Magnitude^d^	4.88 (0.07)	4.92 (0.08)	5.02 (0.10)	4.71 (0.08)	4.60 (0.09)	4.50 (0.09)
Perceived balance of drug benefits and risks^e^		4.50 (0.10)	4.43 (0.12)	4.62 (0.15)	4.57^f^ (0.08)	4.25 (0.10)	4.37 (0.12)
**Intention^g^**							
	Physician interaction	2.81 (0.08)	2.81 (0.10)	2.83 (0.13)	2.48 (0.10)	2.37 (0.10)	2.12 (0.12)
	Search on the Internet	2.39 (0.08)	2.48 (0.09)	2.50 (0.12)	2.36 (0.10)	2.14 (0.09)	2.11 (0.11)
Website skepticism^h^		3.35^f^ (0.07)	3.58^c^ (0.09)	3.24 (0.12)	3.70 (0.09)	3.82 (0.09)	3.69 (0.11)
Perceived balance of website benefit and risk information^i^		5.05 (0.09)	4.83 (0.11)	4.69 (0.16)	5.07^c^ (0.10)	4.85^c^ (0.10)	4.27 (0.16)

^a^Perceived drug risk and efficacy likelihood: 0-100 people. Although transformations of perceived drug risk and efficacy likelihood were used in analyses, the untransformed weighted means are presented here for ease of interpretation.

^b^Perceived drug risk magnitude: 1 (not at all serious) to 6 (very serious).

^c^Significantly different from the control condition; Bonferroni-adjusted for two comparisons with the control condition, *P*<.025.

^d^Perceived drug efficacy magnitude: 1 (help a little) to 6 (help a lot).

^e^Perceived balance of drug benefits and risks: 1 (risks outweigh benefits) to 7 (benefits outweigh risks).

^f^Significantly different from the testimonial condition, *P*<.05.

^g^Intentions: 1 (very unlikely) to 5 (very likely).

^h^Website skepticism: 1 (extremely unlikely) to 7 (extremely likely).

^i^Perceived balance of website benefit and risk information: 1 (more emphasis on risks) to 7 (more emphasis on benefits).

#### High Blood Pressure

We tested the effect of risk prominence and video type condition on the perceived balance of drug benefits and risks. We found a significant effect of video type condition. Participants in the mechanism of action condition thought that the benefits outweighed the risks compared with participants in the testimonial condition (*F*_1,809_=6.25, *P*=.01, *d*=.18).

### Behavioral Intentions

#### Acid Reflux and High Blood Pressure

We tested the effect of risk prominence and video type condition on physician interaction and Internet search intentions. In both the acid reflux and high blood pressure samples, we found no significant effects (*P*>.05).

### Website Skepticism

#### Acid Reflux

We tested the effect of risk prominence and video type condition on website skepticism. We found a significant effect of video type condition and a significant interaction. Participants in the testimonial condition were more skeptical of the website compared with participants in the control and mechanism of action conditions (*F*_1,1063_=5.47, *P*=.02, *d*=.14 and *F*_1,856_=4.14, *P*=.04, *d*=.14, respectively). A significant interaction with risk prominence (*F*_1,856_=4.63, *P*=.03) suggests that this was driven by the low-risk prominence conditions (testimonial, low-prominence condition mean=3.72, SE 0.12; mechanism of action video, low-prominence condition mean=3.25, SE 0.11; testimonial, high-prominence condition mean=3.44, SE 0.13; mechanism of action video, high-prominence condition mean=3.46, SE 0.10).

#### High Blood Pressure

We tested the effect of risk prominence and video type conditions on website skepticism. We found no significant effects (*P*>.05).

### Perceived Balance of Website Benefit and Risk Information

#### Acid Reflux

We tested the effect of risk prominence and video type condition on the perceived balance of website benefit and risk information. The effect of risk prominence was significant; participants in the low-risk prominence condition thought that the website placed more emphasis on benefits compared with participants in the high-risk prominence condition (*F*_1,857_=9.50, *P*=.002, *d*=.21).

#### High Blood Pressure

We tested the effect of risk prominence and video type condition on the perceived balance of website benefit and risk information. We found significant effects when comparing video type conditions with the control condition, and a significant effect of risk prominence. Participants in the mechanism of action and testimonial conditions thought that the website placed more emphasis on benefits compared with participants in the control condition (*F*_1,1037_=17.56, *P*<.001, *d*=.26 and *F*_1,1037_=9.35, *P*=.002, *d*=.19, respectively). Participants in the low-risk prominence condition thought that the website placed more emphasis on benefits compared with participants in the high-risk prominence condition (*F*_1,809_=6.17, *P*=.01, *d*=.17).

## Discussion

### Principal Findings

This study investigated how videos embedded on branded prescription drug websites influence consumers’ knowledge, perceptions, and intentions. We tested websites with no video, with a testimonial, and with an informational video describing the drug’s mechanism of action. Within the testimonial and mechanism of action videos, we manipulated the prominence of risk information, with some risk information included, or not included, in the videos (with risk information always present in the text of the website). The results of this study suggest that embedded videos can affect consumers’ knowledge of drug information but are less likely to affect perceptions and intentions.

We found that including risk information in videos increased participants’ recognition of the risks presented in the videos. However, in some cases, including risk information in videos decreased participants’ recognition of the risks *not* presented in the videos (ie, risks presented in text only). The Communication-Human Information Processing Model states that for risk information to be understood, individuals must first switch their attention to the information and then maintain their attention on the information [35,36]. The videos may capture and maintain their attention; therefore, when videos are presented on websites, the inclusion of risks in the videos can increase the retention of some of those risks. At the same time, it may decrease the retention of risks not mentioned in the video by preventing individuals from switching and maintaining their attention on the text. Participants may assume that all risks are included in the video, and therefore, they may not read the risk information provided only in the website’s text.

Including risks in the video did not affect participants’ retention of the drug’s main benefit, although in the high blood pressure sample, it did decrease the perceived magnitude of drug efficacy. In addition, we found that not including risk information in the videos shifted participants’ views of the website, causing them to believe that the website placed more emphasis on benefits. In most cases, however, this did not translate into increased skepticism of the website; only participants in the acid reflux sample who saw the testimonial without risk information reported more skepticism. These findings suggest that if videos are used, the inclusion of risk information in videos can increase consumers’ knowledge of the risks while not diminishing consumers’ knowledge of the drug’s benefit or making them more skeptical.

We found that the videos had no effect on intentions and a limited effect on risk perceptions; in the acid reflux sample only, including risk information in the videos increased the perceived magnitude of the drug’s risks. Quantifying or personalizing the risks in testimonials may be necessary to change perceptions. These findings are also consistent with previous research demonstrating that consumers consider numerous sources of information about prescription drugs—particularly, health care providers—and that their perceptions of and intentions about specific medications are influenced by multiple factors, including their personal health history, satisfaction with current treatment, and knowledge of others’ experiences with the drugs [37-43]. Consequently, Web videos promoting a prescription drug may not be powerful or persuasive enough to shift individuals’ perceptions and intentions unless they are part of a larger marketing campaign or align with a health care provider’s recommendation to take a medication.

The study samples consisted of individuals who had been diagnosed with one of two health conditions—acid reflux and high blood pressure. Across medical conditions, we saw a similar pattern of effects for risk prominence, such that risk prominence affected risk retention but had little or no effect on benefit retention and perceptions. This provides some confidence that these results would generalize across different medical conditions and different website and video executions. However, the effects of video type condition were not consistent across medical conditions. The two video styles examined in this study—testimonials and mechanism of action videos—were not entirely equivalent, and the executions differed across medical conditions. To be realistic and to mirror actual content on branded drug websites, the videos contained different benefit information. The mechanism of action videos focused on how each fictitious drug worked; the testimonials presented an individual patient’s experience benefiting from the fictitious drug. This distinction means that differences between testimonial and mechanism of action video type conditions in the study’s findings could be caused by multiple factors, such as video style (eg, live action vs animated), video content, or even video duration. The benefit retention results reflect this; in the acid reflux sample, the mechanism of action video decreased the retention of the drug’s benefits, whereas it increased retention in the high blood pressure sample. Future research should standardize content within testimonials and mechanism of action videos to determine whether differences are attributable to visuals or content. This would provide a less realistic, but more controlled, setting in which these concepts can be tested.

### Limitations

This study was a controlled experiment with realistic stimuli, large sample sizes, and high statistical power. Nevertheless, the study has several limitations that should be considered when interpreting the findings. First, the study samples were limited to individuals with household broadband Internet access and, thus, may contain a higher proportion of white, older, and more educated individuals than the US adult population. The study was also limited to participants with two illness conditions and did not include individuals without health issues. One avenue for future research is to test these concepts in a different sample. For instance, future research could focus on individuals actively seeking prescription treatments (eg, newly diagnosed and dissatisfied with current treatment). Future research should also examine whether key differences between these two illness conditions (eg, symptomatic vs asymptomatic, many nonprescription treatment options vs few nonprescription treatment options) explain why certain effects were present in only one population.

Individuals may be passively exposed to television and print DTC advertising, whereas individuals who visit DTC websites may be actively seeking information about treatments. Thus, participants in our study may have had less interest in and paid less attention to the study DTC website compared with individuals actively seeking information on the Internet. Conducting future research with individuals actively seeking prescription treatments would partially address this limitation as well.

### Conclusions

Our results reflect the caution in using testimonials urged by Elwyn and colleagues [27]. Including a video on a prescription drug website can affect website credibility (for instance, by increasing skepticism and changing the perceived balance of information on the website). It can also enhance or detract from consumers’ knowledge of the drug’s benefits and risks. When videos are used on prescription drug websites, the inclusion of risk information in the videos can lead to greater knowledge of the product’s important risk information. Thus, “fair balance” may be enhanced by including risk information in website videos.

## Abbreviations

ANOVA: analysis of variance
DTC: direct-to-consumer
FDA: Food and Drug Administration
